# *Cryptococcus gattii* strains with a high phagocytosis phenotype by macrophages display high pathogenicity at the early stage of infection *in vivo*: High phagocytosis Cg displays early pathogenicity

**DOI:** 10.3724/abbs.2023250

**Published:** 2023-10-26

**Authors:** Chen Yang, Wanjun Shen, Lifeng Wang, Xuelei Zang, Yemei Huang, Hengyu Deng, Yangyu Zhou, Mei Xie, Xinying Xue, Dingxia Shen

**Affiliations:** 1 Department of Laboratory Medicine the First Medical Centre Chinese People’s Liberation Army (PLA) General Hospital Beijing 100853 China; 2 State Key Laboratory of Kidney Disease Department of Nephrology Chinese People’s Liberation Army (PLA) General Hospital Beijing 100853 China; 3 Department of Respiratory and Critical Care Beijing Shijitan Hospital Capital Medical University Peking University Ninth School of Clinical Medicine Beijing 100089 China; 4 School of Clinical Medicine Weifang Medical University Weifang 261053 China; 5Department of Respiratory and Critical Care Chinese People’s Liberation Army (PLA) General Hospital Beijing 100853 China

**Keywords:** *Cryptococcus gattii*, macrophage, phagocytosis, pathogenicity, granuloma

## Abstract

*Cryptococcus gattii* (Cg) is a facultative intracellular pathogen that can replicate and disseminate in mammalian macrophages, causing life-threatening cryptococcosis in both immunocompetent and immunocompromised individuals. *Cryptococcus-*macrophage interactions are crucial for cryptococcosis prognosis. However, the relationship between Cg pathogenicity and phagocytosis by macrophages has not yet been investigated in depth. In this study, a series of *in vitro* and *in vivo* experiments were conducted to investigate the interaction between macrophages and Cg. Flow cytometry was used to detect the phagocytic phenotypes of the Cg strains within macrophages. Scanning electron microscopy, transmission electron microscopy, and immunofluorescence were used to observe phagocytosis and proliferation, respectively. Survival and lung fungal burden tests were also performed. Our results show that Cg cells display different phagocytosis phenotypes, which are independent of the molecular type. Within macrophages, the high phagocytosis phenotype (HP) strains obtain higher intracellular proliferation than the low phagocytosis phenotype (LP) strains. At the early stage of infection *in vivo*, HP-inducing permissive granulomas within the lungs seldom limit the dissemination of cryptococci. In addition, HP strains could inhibit the formation of M1-type macrophages, proliferate intracellularly and disseminate extracellularly, and cause hypoxia induced by mucus and acidic polysaccharide accumulation in pulmonary alveoli much earlier than LP strains *in vivo*. Our work reveals that Cg displays diverse interactions with macrophages, which may enhance our understanding of the pathogenicity of this life-threatening pathogen.

## Introduction

In 2022, the WHO issued a list of fungal priority pathogens, of which the *Cryptococcus* genus has received intensive attention [1]. *Cryptococcus* is a lethal pathogen that seriously threatens public health [2]. *Cryptococcus neoformans* (Cn) and *C*. *gattii* (Cg) are the two main pathogens causing cryptococcosis. In immunocompromised individuals, Cn frequently causes cryptococcal meningitis, an extremely life-threatening disease [3]. Unlike Cn, Cg targets both the respiratory and central nervous systems and can infect both immunocompetent and immunocompromised individuals, with a mortality rate of nearly 33% [ 2, 4, 5].

In response to invasion by *Cryptococcus*, the host immune system develops a significant and intricate immunological mechanism [6]. *Cryptococcus* may remain dormant for years within the host and therefore induce specific immunity [7]. Cryptococci, as yeast cells or spores, enter the alveoli of the host through the respiratory tract and are then exposed to the immune environment of the host, with phagocytes serving as the first line of defense. Among the various phagocytes, macrophages are crucial in immunological functions, including recognizing capsule polysaccharides, phagocytosis, killing, generation of cytokines and chemokines, and presenting antigens [ 8, 9]. Several previous studies have revealed that macrophages not only maximize the killing of *Cryptococcus* but also assist in allowing the immune escape of *Cryptococcus*. Macrophages have been found to provide an ecological niche for *Cryptococcus* to replicate intracellularly, which could limit the impact of extracellular antifungal medications [ 10, 11]. Macrophages are also involved in transporting *Cryptococcus* through the blood-brain barrier via the Trojan mechanism [12]. Macrophages may switch their phenotypes to the classical activated M1 type, which acts in the early containment of *Cryptococcus*, and the alternatively activated M2 type, which promotes fungal persistence [13].

Numerous previous studies have highlighted the crucial virulence roles of *Cryptococcus* phagocytosis and proliferation within macrophages in cryptococcosis [ 11, 14, 15]; however, compared to Cn, Cg received less attention and appeared to have a lower incidence but caused outbreaks and even infected people with normal immunity. Cn with high uptake by macrophages was previously reported to be correlated with central nervous system (CNS) fungal burden and poor clinical outcomes [11]. It is still unknown whether the phagocytosis phenotypes of Cg and Cn by macrophages are consistent or whether the phagocytosis phenotype is related to the Cg cryptococcosis outcome. In addition, outbreak strains of Cg (VGIIa) have been demonstrated to increase the proliferation rate within host macrophages, which is positively correlated with virulence in a mouse model of cryptococcosis [16]. Whether a similar conclusion can be drawn among the nonoutbreak Cg strains isolated in China is worth further investigation.

In this study, we conducted a series of *in vitro* and *in vivo* experiments to investigate the interactions between macrophages and Cg. Our results suggested that Cg strains with a high phagocytosis phenotype within macrophages display high pathogenicity at the early stage of infection *in vivo*.

## Materials and Methods

### Ethics statement

The study was approved by the Animal Experimental Ethics Committee of Beijing Shijitan Hospital [Protocol Code: sjtky11-1x-2020 (20)]. Five mice per group were housed in filtered cages with top ventilation and a 12/12 h light/dark cycle, with free access to food and water. Mice were intraperitoneally anaesthetized with 100 mg/kg ketamine and 10 mg/kg xylazine prior to the study of mortality. The mice were examined twice daily for any signs of distress, which were determined by weight loss, weakness, or inability to feed or drink. The mice were humanely sacrificed as soon as signs of distress appeared.

### Cryptococcus strains

A total of 43 clinical *Cryptococcus* spp. isolates were collected between 2011 and 2017 in China, of which 28 Cg isolates were differentiated from 15 Cn isolates on canavanine glycine bromothymol blue (CGB) agar [17] and reidentified by matrix-assisted laser desorption ionization time-of-flight mass spectrometry (MALDI-TOF MS) (Bruker Daltonik, Bremen, Germany). Furthermore, 21 VGI strains and 7 VGII strains of Cg were identified by molecular typing using URA5-RFLP analysis based on multilocus sequence typing (MLST) [18]. Three Cg reference strains (R265, R272, and WM276) were used as control strains in all experiments. All strains were preserved in 20% glycerol at ‒80°C and recovered in Sabouraud dextrose agar (SDA) plates (Autobio Diagnostics, Zhengzhou, China) at room temperature (RT). Prior to *in vitro* and *in vivo* experiments, a single colony of cryptococci on SDA plates was transferred into Sabouraud dextrose broth (SDB) (Comagal Microbial, Shanghai, China) and incubated at a shaking speed of 200 rpm at 37°C for 24 h [ 11, 19].

### Macrophage culture

Murine-derived RAW264.7 macrophages were selected as the phagocytosis model and routinely incubated at 37°C in a 5% CO _2_ atmosphere in Roswell Park Memorial Institute (RPMI) 1640 medium with L-glutamine (Gibco, Carlsbad, USA) supplemented with 10% fetal bovine serum (FBS; Gibco) and 1% penicillin-streptomycin (Gibco). Prior to infection, cells were seeded in 24-well plates (Sigma-Aldrich, St Louis, USA) at a density of 1.5×10 ^5^ cells per well and cultured overnight. To control for variations among passages, the cell batches were limited to three passages.

### Activation of macrophages and opsonization of cryptococci

Unless otherwise noted, in our experiments, macrophages were infected with cryptococci at a multiplicity of infection (MOI, cryptococci: macrophage) of 10. To achieve a better phagocytic state, macrophages were activated with 0.5 μg/mL lipopolysaccharides (MedChemExpress, Shanghai, China) and 10 ng/mL IFN-γ (MedChemExpress) to induce M1 polarization for 16 h before infection. Cryptococci were collected by centrifugation from the 24-h SDB culture, washed twice with phosphate buffered solution (PBS), and opsonized with 1 μg/mL mAb 18B7 (MABF2069; Sigma-Aldrich), which was directed against the cryptococcal capsular polysaccharide glucuronoxylomannan (GXM), for 60 min at RT [11]. To verify whether the monoclonal antibody stained the capsules of all isolates in the same way, a double agar gel immunodiffusion test (DID)-based immunoprecipitation was performed. The capsular polysaccharide, including antigen GXM, was harvested from the supernatant of overnight cultures grown in yeast nitrogen base (YNB) medium (Sigma-Aldrich) at 30°C with a shaking speed of 150 rpm, as described previously [20]. Briefly, to eliminate any remaining cryptococci, the culture supernatant was filtered through 0.45 μm pore size filters (Sigma-Aldrich). The polysaccharides were precipitated by the addition of 10% (w/v) sodium acetate and 2.5 volumes of ethanol, dried, and dissolved in water [21]. DID between the monoclonal antibody 18B7 (1 μg/mL) in the central well and the polysaccharide antigen isolated from each organism was performed according to the specifications of Kobayashi *et al*. [22]. The attachment of mAb 18B7 to GXM in the polysaccharides of each isolate was determined by three biological replicate tests. For each test, positive (Cg R265) and negative controls ( *Candida albicans*; ATCC 10231) were included.

### Immunostaining and observation of phagocytosis and proliferation

Activated macrophages (1×10 ^5^ cells) were cultured to adhere to coverslips pretreated with 0.01% polylysine (Solarbio, Beijing, China) in 24-well plates supplemented with 500 μL serum-free RPMI 1640 medium and then exposed to 500 μL suspension of opsonized cryptococci containing 1×10 ^6^ yeasts. The mixture was then incubated at 37°C with 5% CO _2_. Unengulfed extracellular cryptococci were removed with sterile PBS and extensively washed three times. Two time-points were set for this experiment: 2 h and 18 h. After reaching the scheduled time, the samples were fixed in 4% paraformaldehyde for 30 min at RT, washed three times with PBS, and permeabilized in 0.25% Triton X-100 for 20 min at RT. Blocking was performed using 10% normal goat serum (Solarbio) for 30 min at RT. Staining with primary antibodies: mouse anti-GXM mAb 18B7 (Sigma-Aldrich) and rabbit anti-CD11b antibody (ab184308; Abcam, Cambridge, UK), was performed at 4°C and 1:500 in 10% normal goat serum-PBS solution overnight. The samples were washed thrice with PBS. Secondary antibody staining [goat anti-mouse IgG H&L (DyLight ^®^ 488) (ab96879; Abcam) and goat anti-rabbit IgG H&L (Alexa Fluor ^®^ 647) (ab150079; Abcam)] was performed for 60 min at RT at 1:1000 in 10% normal goat serum-PBS. The unbound secondary antibodies were washed three times with PBS. Slides were mounted using a mounting medium containing DAPI (ab104139; Abcam). Samples without challenging cryptococci served as the negative controls. The specimens were analyzed by confocal microscopy (NIKON Eclipse Ti, Tokyo, Japan). Approximately 100 microscopic fields were observed, and 20 images were obtained from each isolate in three biological replicates.

### Phagocytosis assay by flow cytometry

The number of macrophages infected with FITC-cryptococci was quantified by fluorescence-activated cell sorting (FACS) using flow cytometry as previously described [ 23, 24]. Briefly, before coincubation, the cryptococci were labelled with 0.5 mg/ml fluorescein isothiocyanate (FITC; Sigma-Aldrich) in 0.1 M NaHCO _3_ (Sigma-Aldrich) at 30°C for 1 h. Unbound FITC was washed off with PBS three times or until the supernatant was clear. FITC-labelled cryptococci were then opsonized with 1 μg/mL mAb 18B7, and the final concentration was determined to be 1×10 ^6^ yeast/mL in PBS. These labelled strains were further analyzed by flow cytometry (BD Fortessa, Franklin Lakes, USA) to ensure that the proportion of FITC-labelled cryptococci was greater than 0.99.

The activated macrophages were precultured to adhere to 6-well plates in serum-free medium for 30 min and cocultured with 10-fold opsonized FITC-labelled cryptococci at 37°C in a 5% CO _2_ atmosphere for 2 h. The extracellular cryptococci, which were not engulfed, were removed by gentle washing with PBS three times. Next, macrophages were detached with TrypLE ^TM^ solution (Gibco), collected by centrifugation (230 *g*, 5 min), and fixed with 500 μL of 70% ethanol. The integrity of the macrophages was detected by light microscopy. Superficial residual fluorescence on macrophages was quenched by adding 100 μL Trypan Blue (0.04%) to eliminate interference fluorescence. Flow cytometry (BD Fortessa) was utilized to separate the infected (FITC-cryptococci) macrophages from the uninfected (FITC-negative) cells. The percentage of the FITC-positive cell group of the total cell population, defined as the phagocytic index here, was assessed using FlowJo software (version 10.8.1; FlowJo, Ashland, USA). The macrophage groups were gated using a combination of forward scatter (FSC) and sideways scatter (SSC). The FITC-positive cell group was then gated according to a blank control (mock-infected macrophages) and the positive control (macrophages infected with FITC-R265). For each sample, 10,000 macrophages were counted, and the average phagocytic indexes were obtained from three independent biological replicate tests.

### Sample preparation for scanning electron microscopy (SEM) and transmission electron microscopy (TEM)

Macrophages were challenged with cryptococci for 90 min in sample preparation for SEM, as described above for sample preparation for immunostaining. Next, the macrophages were fixed with 2.5% (v/v) glutaraldehyde (Solarbio), incubated with 1% osmium tetroxide, and dehydrated with an ascending ethanol series. After dehydration and critical point drying, the specimens were coated with gold and analyzed by SEM (Hitachi SU8020, Tokyo, Japan). Approximately 100 microscopic fields were observed, and 15 images were obtained for each isolate. The number of cryptococci adhering to or captured by 100 macrophages from the triple tests was counted, and the average cryptococci number per macrophage was then calculated for each isolate.

For TEM sample preparation, the coculture mixture was incubated for 2 and 18 h. To obtain a visible cell cluster, we enlarged the final volume to 3.0 mL for TEM rather than 1.0 mL for the intracellular cryptococcal survival assay, but the proportion of components and MOI were the same. Phagocytosis was stopped by placing the culture plates on ice. Macrophages were carefully washed thrice with PBS, detached by TrypLE, and collected by centrifugation (230 *g*, 5 min). The pellet was fixed in 2.5% glutaraldehyde for 120 min and washed with 0.1 M phosphate buffer (PB) for 5 min three times. The cell pellet was dehydrated in a graded ethanol series and embedded in Eponate-12. Ultrathin sections were stained with uranyl acetate and lead citrate and examined using TEM (JEM-1400; JEOL, Tokyo, Japan). The observations and images obtained were the same as those obtained by SEM. Approximately 100 cells were observed, and 15 images were obtained for each isolate. Strains in both phagocytosis groups were analyzed and subjected to three separate biological replicates.

### Intracellular cryptococcal survival and proliferation assay *in vitro*

All *Cryptococcus* strains were tested for intracellular cryptococcal load (ICL) and intracellular proliferation rate (IPR). Activated macrophages and opsonized cryptococci were prepared as mentioned in the preparation of the macrophage-cryptococci interaction assay. Before infection, RAW264.7 macrophages (1×10 ^5^ cells) were cultured to adhere to 24-well flat-bottom plates in 500 μL serum-free medium for 30 min. Then, 500 μl cryptococcal suspension containing 1×10 ^6^ yeast cells per well was added. The coculture mixture was incubated at 37°C with 5% CO _2_. After a 2-hour infection, the macrophages were gently and extensively washed three times with PBS to remove extracellular cryptococci. Three time points were set in this experiment: 2, 18, and 24 h. At 2 h postinfection (hpi), macrophages were lysed with 500 μL of sterile water for 15 min at 37°C to release intracellular cryptococci. The complete lysis of macrophages was confirmed by microscopic examination. Similarly, the cells were lysed at 18 and 24 hpi, and intracellular cryptococci were counted. The suspension was diluted 100-fold with PBS. Then, 100 μL of diluted suspension was plated on an SDA medium plate. Colony-forming units (CFUs) were enumerated after a two-day incubation, namely, the ICL. The IPR of *Cryptococcus* was calculated from the ICL at 18 hpi or 24 hpi divided by the ICL at 2 hpi. Each isolate was subjected to three independent biological replicate tests, and the average values were obtained. The results of each group are presented as the mean±standard deviation (SD).

### Murine model of *Cryptococcus* inhalation infection

All isolates grouped by previous phagocytosis assays were tested for their virulence *in vivo*. Strains were cultured overnight at 37°C in SDB medium and washed twice with sterile PBS after centrifugation, and the final concentration was adjusted to 2×10 ^6^ yeast/mL. Female C57BL/6 mice aged 8–10 weeks were anaesthetized with 100 mg/kg ketamine and 10 mg/kg xylazine. Each mouse was then slowly administered 50 μL of fungal suspension in the nasal cavity [25]. Ten mice from the HP and LP groups were infected with each isolate, and a survival test was performed. The death of the mice was observed and recorded twice daily for 90 days.

Meanwhile, five mice in each strain group were tested for fungal load and sacrificed 14 d postinfection. Lungs from dissected mice were placed in 15 mL centrifuge tubes containing 4 mL PBS. The tissue was crushed and homogenized in a biosafety cabinet. The tissue suspension was 100-fold diluted with PBS, and 100 μL was plated on the SDA medium plate. The culture was set at 30°C, and CFUs were counted after a two-day incubation. The average CFUs of each isolate were then calculated, and the lung fungal loads between the HP and LP groups were compared.

### Pathological experiments on the lungs of mice infected with Cg

Six mice per isolate from the HP and LP groups were used for pathological studies. Three mice per isolate were euthanized 14 days postinfection (dpi) or when any signs of distress were observed within 14 days. All remaining surviving mice were euthanized at the end of the experiment (90 days) or when they showed distress from 14 to 90 dpi. Dissected lungs were immediately fixed in formalin, embedded in paraffin, and sectioned. To better understand the relationship between the host and *Cryptococcus*, adjacent sections of the most apparent infective lesions were chosen and stained with hematoxylin-eosin (HE), periodic acid-Schiff (PAS), and Alcian blue (AB) according to previous studies [ 26, 27]. PAS staining localizes to the cell wall of cryptococci, while AB staining interacts with the acid polysaccharide in the capsule [ 26, 27].

Multiple immunofluorescence (mIF) was used to observe the distribution of M1-type macrophages during cryptococcosis. Based on the phagocytic indexes of all the Cg isolates, the top nine strains and the last nine strains were chosen to represent Cg’s high and low phagocytosis groups, respectively. Briefly, the sections were dewaxed and dehydrated using an ascending ethanol series. Antigen retrieval was accomplished using sodium citrate, endogenous peroxidase was inhibited using hydrogen peroxide, and nonspecific protein-binding sites were blocked with 3% bovine serum albumin (BSA). Next, the slices were incubated overnight with diluted rabbit primary antibody CD86 [28] (1:3000; Servicebio, Wuhan, China) at 4°C and then incubated in the dark with horseradish peroxidase (HRP)-labelled goat anti-rabbit secondary antibody at 1:500 (Servicebio), followed by the corresponding tyramide signal amplification reagent (TSA; iF488-tyramide; 1:500; Servicebio). Sections were then mounted using mounting medium containing DAPI. Finally, microscopic observations, image acquisition, and analysis were performed. The site of infection was first determined in HE sections, the number of cryptococci was counted, and then the number of M1-type macrophages (M1-MØ) was counted in the corresponding area of immunofluorescence sections. In each section, one area where cryptococci were gathered was randomly selected, and the number of macrophages was normalized per 100 cryptococci. The relative numbers of M1-MØ induced by each strain at the early and late stages were averaged and then compared between the HP and LP groups composed of different isolates. Lung sections from uninfected mice served as a negative control.

### Statistical analysis

All data were analyzed using SPSS software (version 19.0; IBM, New York, USA). Continuous variables between the two groups were assessed according to whether the data were normally distributed using the Student’s *t* test or Mann-Whitney U test. The differences among the three groups were determined using a one-way analysis of variance (ANOVA). All statistical tests were two-sided. *P*<0.05 was considered statistically significant. Survival in murine infection analysis was compared using the log-rank (Mantel-Cox) test in GraphPad Prism software. Associations between the intracellular proliferation rate and mouse half-life time (ST50), as well as phagocytic index, were analyzed using Pearson’s correlation and linear regression analyses. ImageJ (version 1.8.0; NIH, Bethesda, USA) was used for cryptococci and M1-macrophage counting in the lung sections, and GraphPad Prism was used for plotting.

## Results

### Macrophage phagocytosis of VGI, VGII, and Cn was observed by confocal microscopy.

A total of 43 clinical *Cryptococcus* spp. isolates were collected in China, of which 28 Cg isolates were differentiated from 15 Cn isolates on canavanine glycine bromothymol blue (CGB) agar and reidentified by matrix-assisted laser desorption ionization time-of-flight mass spectrometry (MALDI-TOF MS). Furthermore, 21 strains of Cg VGI (75%, 21/28) and 7 strains of VGII (25%, 7/28) were identified by molecular typing using URA5-RFLP analysis based on multilocus sequence typing (MLST). Three Cg reference strains (WM276 [VGI], R265 [VGII], and R272 [VGII]) were used as control strains.

**Figure 1:**
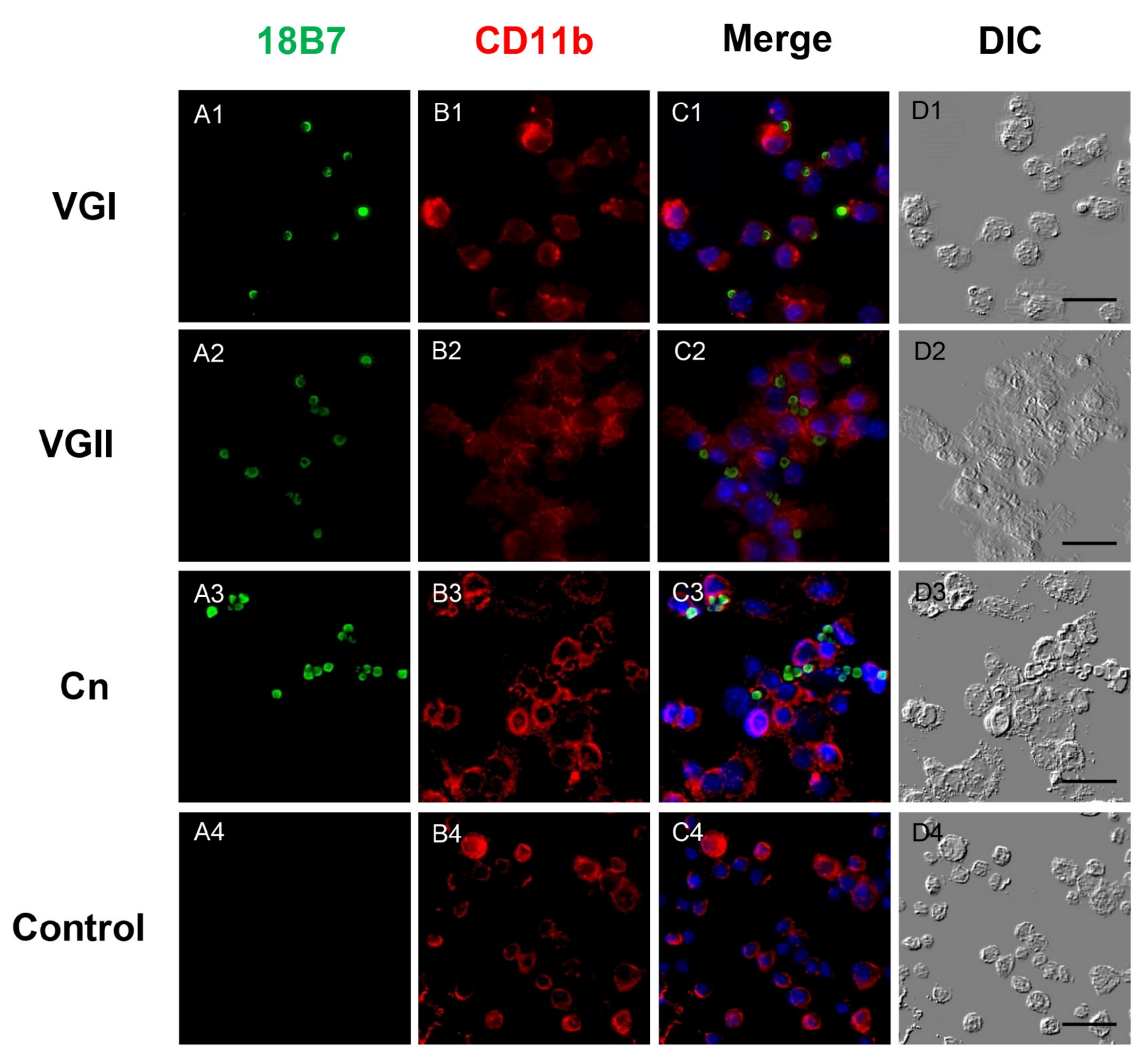
Figure 1 Phagocytosis of VGI, VGII, and Cn by macrophages was observed by immunofluorescence Phagocytosis was observed in all our Cryptococcus strains. Macrophages challenged by VGI (22 strains, A1‒D1), VGII (9 strains, A2‒D2), and Cn (15 strains, A3‒D3) were stained for cryptococci (GXM, green, A1‒A3), macrophages (membrane, red, B1‒B3) and nuclei (DAPI, blue, C1‒C3). The merged images (C1‒C3) and DIC images (D1‒D3) illustrated that cryptococci of VGI, VGII, and Cn could all be phagocytosed by macrophages. Samples without cryptococcal infection served as negative controls (A4‒D4). Images are representative of three independent tests that were carried out with similar outcomes. GXM, glucuronoxylomannan; DIC, differential interference contrast. Scale bar: 20 μm.

To directly observe whether macrophages can engulf different species of *Cryptococcus*, confocal microscopy was used to observe the murine macrophage-like cell line RAW264.7 and cryptococci in coculture. RAW264.7 cell membrane and *Cryptococcus* capsule GXM were specifically labelled with primary antibodies anti-CD11b and monoclonal antibody (mAb) 18B7, respectively. To ensure that the monoclonal antibody stains the capsule of all isolates in the same way and avoid Fcγ receptor-dependent phagocytosis being affected by 18B7 decreasing attachment to the capsule, the assessment of 18B7 attachment to GXM in polysaccharides of each isolate needs to be determined. A double agar gel immunodiffusion test (DID) demonstrated that 18B7 could effectively bind to the capsule of each isolate, yielding precipitin lines (data not shown). Phagocytosis of all our *Cryptococcus* strains was tested by immunofluorescence, and images are representative of three separate studies that were carried out with similar outcomes. As Figure 1 illustrates, at 2 hpi, VGI, VGII, and Cn could all be captured by macrophages or even phagocytosed.

### Cg displayed diverse phagocytic index by macrophage

**Figure 2:**
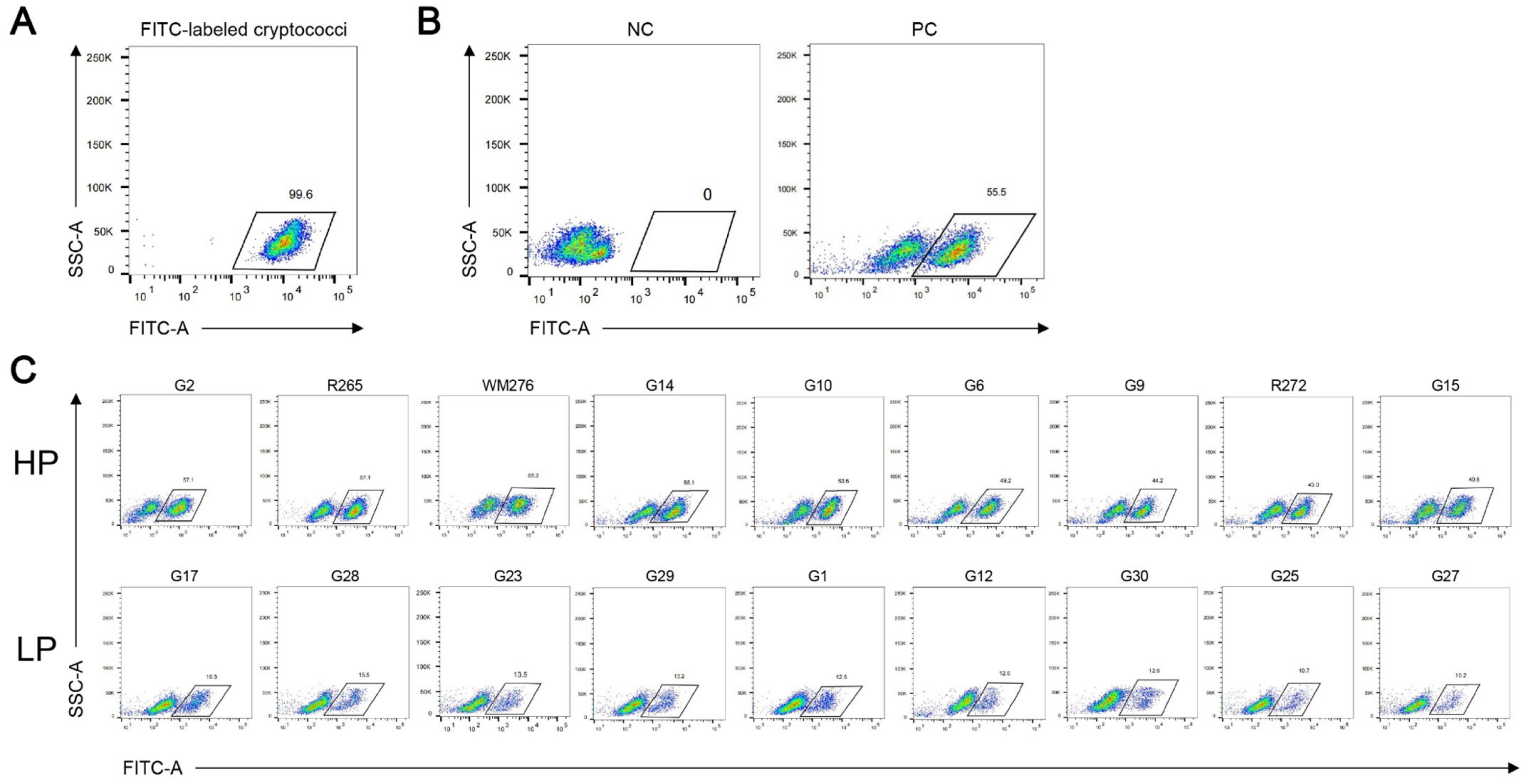
Figure 2 Cg displayed diverse phagocytosis phenotypes by macrophages according to flow cytometry (A) The FITC labelling rate of cryptococci was greater than 99%. (B) Uninfected macrophages as a negative control showed weak FITC signals, and the infected macrophages with strong fluorescence signals could be clearly distinguished from the unphagocytosed cell population in the positive control. (C) The proportion of macrophages infected with cryptococci (FITC-positive) was defined as the phagocytic index. Flow cytometry plots of the top nine strains in the high phagocytosis group and the last nine strains in the low phagocytosis group are illustrated. These results are representative of three independent biological replicate tests. NC, negative control. PC, positive control. HP, high phagocytosis. LP, low phagocytosis.

The phagocytic index of Cg by macrophages was assessed by flow cytometry. Very little lysis was observed during the collection of macrophages. When stained cryptococci were assessed for FITC status by flow cytometry, more than 99% were positive for FITC ( Figure 2A). Phagocytosis by macrophages with strong FITC fluorescence was measured and could be clearly distinguished from the unphagocytosed cell population ( Figure 2B). We first compared the phagocytic index according to Cg’s molecular typing, and no significant difference was observed between VGI and VGII ( Supplementary Figure S1; *P*=0.1113). We divided isolates into high- and low-phagocytosis (HP & LP) groups based on the median phagocytic index (29%; Supplementary Table S1). Plots of the top nine and last nine isolates are illustrated ( Supplementary Table S1 and Figure 2C). Among these tested Cg strains, the HP and LP groups were both composed of VGI- and VGII-type strains. HP contained 10 VGI- and six VGII-type strains, and LP had 12 VGI- and three VGII-type strains.

### Phagocytosis was observed by SEM

**Figure 3:**
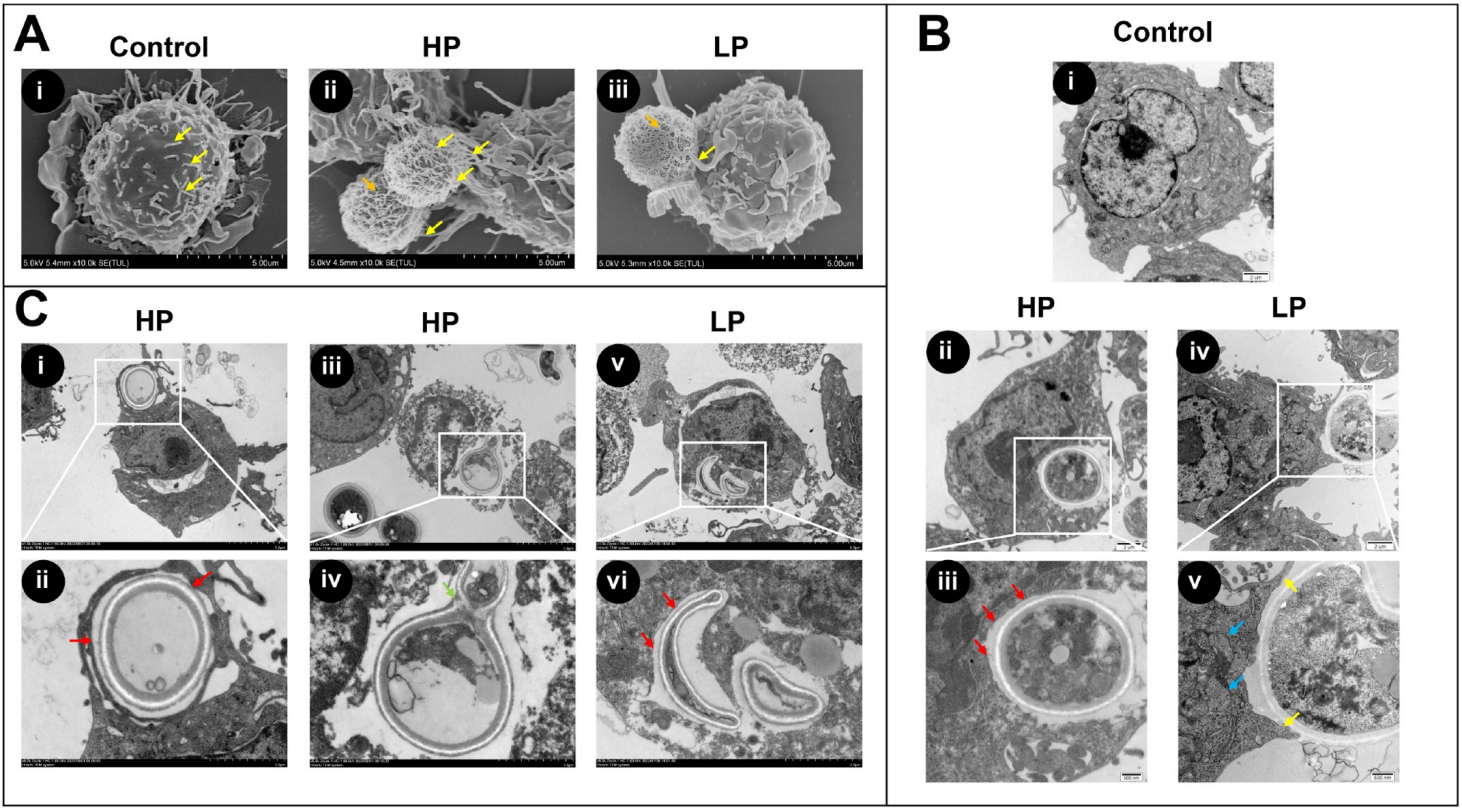
Figure 3 Phagocytosis and the fate of Cg with different phagocytosis phenotypes within macrophages were observed under electron microscopy (A) Different phagocytosis phenotypes of Cg by macrophages under SEM. Macrophages with cryptococci uninfected (blank control) were round-like with many short filopodia (panel i). Macrophages grasped HPs more actively (panel ii) than LPs (panel iii). The filopodia and the polysaccharide fibres are indicated by yellow and orange arrows, respectively. Scale bar: 5 μm. SEM, scanning electron microscopy. (B) Different phagocytosis phenotypes of Cg within macrophages under TEM. Macrophages with cryptococci uninfected (blank control) were morphologically intact with numerous phagocytic vesicles (panel i). Cg HP was surrounded by a phagosomal membrane (red arrows, panels ii and iii), and LP was engulfed by macrophages with filopodia stretching out (yellow arrows, panels iv and v). Scale bar: 2 μm (panel i), 1 μm (panels ii and iv), and 500 nm (panels iii and v). TEM, transmission electron microscopy. (C) Different fates of Cg HP and LP within macrophages were observed by TEM. HP could be released by nonlytic exocytosis (phagosomal membrane as red arrows, panels i and ii) or apoptotic macrophages (germination as green arrow, panels iii and iv). LP was confined within phagosomes (red arrows, panels v and vi). Scale bars, 5 μm (panels i, iii, and v) and 2 μm (panels ii, iv, and vi). In the SEM and TEM analyses, approximately 100 cells were observed, and 15 images were obtained for each isolate. All isolates in the two groups were involved in experiments, and the images are representative of three independent experiments conducted with similar results.

To visualize the capture of cryptococci by macrophages, we used SEM to observe the coculture at 1.5 hpi. Macrophages not challenged with cryptococci as a blank control displayed a round-like morphology with a lightly folded membrane and many short filopodia ( Figure 3A-i, yellow arrows) after being activated by IFN-γ and lipopolysaccharides. Figures 3A-ii and 3A-iii showed that HP and LP cryptococci were captured by macrophages and confined around them. Compared to confronting LP isolates, macrophages stretched out filopodia to grasp HP isolates more actively. Meanwhile, a semiquantitative analysis showed that the number of cryptococci captured by macrophages differed between the two groups. The number of HP and LP cryptococci captured per macrophage was 1.30±0.31 and 0.93±0.21 ( *P*=0.0315), respectively. In addition, HP and LP cryptococci were both observed to form curly polysaccharide fibers on the surface, which were part of the polysaccharide capsule, protecting fungal cells against phagocytosis by macrophages [ 15, 29] ( Figures 3A-ii and 3A-iii, orange arrows indicate fibers).

### Phagocytosis and the fate of Cg within macrophages were observed by TEM

TEM observations demonstrated that the uninfected macrophages were morphologically intact and showed many intracellular phagocytic vesicles and an intact cell membrane with obvious pseudopods ( Figure 3B-i). At 2 hpi, HP isolates were completely engulfed by macrophages with an intact phagosomal membrane surrounding them ( Figures 3B-ii and 3B-iii, red arrows). Meanwhile, phagocytosis of most LP isolates has not yet been completed ( Figure 3B-iv). The structure-deformed macrophages stretched out filopodia ( Figure 3B-v, yellow arrows) to swallow the cryptococci. On the site of the macrophage where phagocytosis occurred, a large number of phagocytic vesicles (blue arrows) gathered for subsequent pathogen engulfment and internalization. Additional TEM images of HP and LP isolates with macrophages at 2 hpi are illustrated in Supplementary Figure S2.

At 18 hpi, different fates of Cg HP and LP cryptococci were observed within macrophages. Figures 3C-i and 3C-ii showed that the macrophages were structurally intact and nonapoptotic. HP cryptococci could be released into the extracellular space by nonlytic exocytosis with phagosomal membranes surrounding it (indicated by red arrows). Figures 3C-iii and 3C-iv showed that HP cryptococci were released extracellularly by apoptotic macrophages, with structural destruction of macrophages and the release of large amounts of lysosomal enzymes. Figures 3C-v and 3C-vi demonstrated that the phagosomes confined the collapsed organism in the LP group, preventing its multiplication or escape (red arrows indicate the phagosomal membrane).

### Cg strains with diverse phagocytosis phenotypes displayed differential intracellular cryptococcal loads (ICLs) and intracellular proliferation rates (IPRs) within macrophages

**Figure 4:**
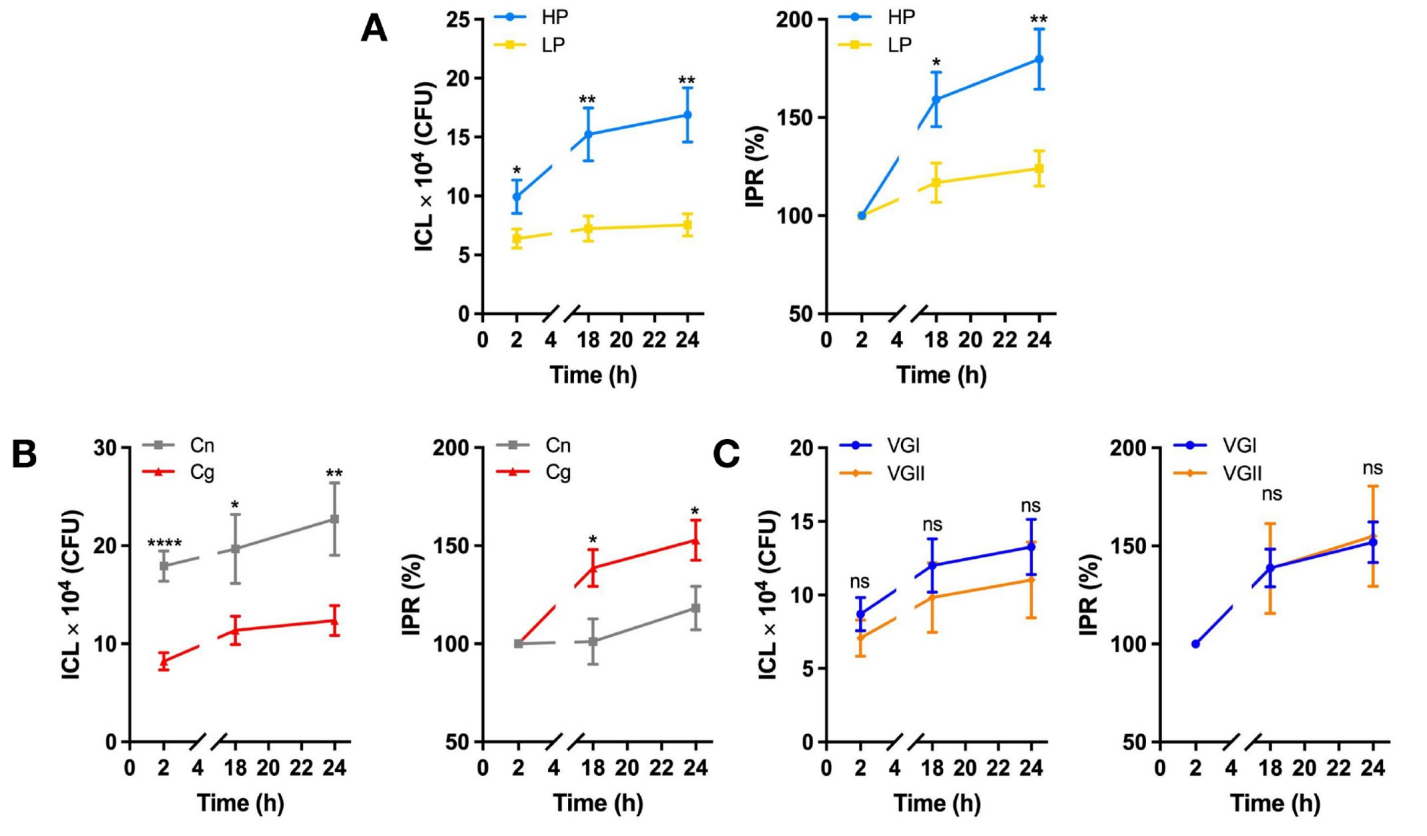
Figure 4 Cg strains with diverse phagocytosis phenotypes displayed differential ICLs and IPRs The ICL and IPR of the HP group were significantly higher than those of the LP group (panel A). The ICL of Cn was greater than that of Cg, while the IPR of Cg was greater than that of Cn (panel B). No significant difference existed between VGI and VGII in ICL or IPR (panel C). Each isolate underwent three independent biological replicate tests, and average values were obtained. The results were from different isolates per phagocytosis group and expressed in CFUs and percentage, and all values were represented as the mean±SD. Differences were considered significant when P<0.05. ns, no significant difference. ICL, intracellular cryptococcal load. IPR, intracellular proliferation rate. hpi, hour postinfection. CFUs, colony-forming units. SD, standard deviation.

To verify whether the intracellular proliferation of Cg cells differ between different phagocytosis phenotypes, ICL and IPR were introduced for quantitative evaluation. Murine-derived RAW264.7 macrophages were cocultured with strains in each group, and the intracellular cryptococcal load was measured at different time points. By counting the colony-forming units (CFUs), we found that the ICL of the HP group was significantly higher than that of the LP group at 2, 18, and 24 hpi ( Figure 4A; *P*=0.0419, *P*=0.0055, *P=*0.0015, respectively). The IPR of HP at 18 and 24 hpi was also significantly higher than that of LP ( Figure 4A; *P*=0.0215, *P*=0.0036). Furthermore, a positive correlation between the phagocytic index and IPR of Cg within macrophages was determined ( Supplementary Figure S3; *P*=0.0030 for IPR-18 hpi, R ^2^=0.2652; *P*=0.0003 for IPR-24 hpi, R ^2^=0.3747. Linear regression, *n*=31).

We also wondered whether a difference exists in intracellular proliferation among different *Cryptococcus* species. Similar to the colony counting methods described above, we found that while the ICL of Cn at the three monitoring time points was greater than that of Cg ( Figure 4B; *P*<0.0001, *P*=0.0127, *P*=0.0035, respectively), the IPR of Cg at 18 and 24 hpi was greater than that of Cn ( Figure 4B; *P*=0.0218, *P*=0.0434). Furthermore, analysis of the Cg subtypes showed no significant differences between VGI and VGII at each monitoring time point, whether ICL ( Figure 4C; *P=*0.4115, *P*=0.5069, *P*=0.5993, respectively) or IPR ( Figure 4C; *P*=0.6229, *P*=0.7813). These data suggested that the Cg strains with a high phagocytosis phenotype were more proliferative within macrophages than those with a low phagocytosis phenotype. Cg was demonstrated to have stronger intracellular proliferation than Cn in our study. Detailed data can be found in the Raw Data Sheet of the Supplementary Material.

### Virulence differed between the Cg HP and LP strains in the murine model

**Figure 5:**
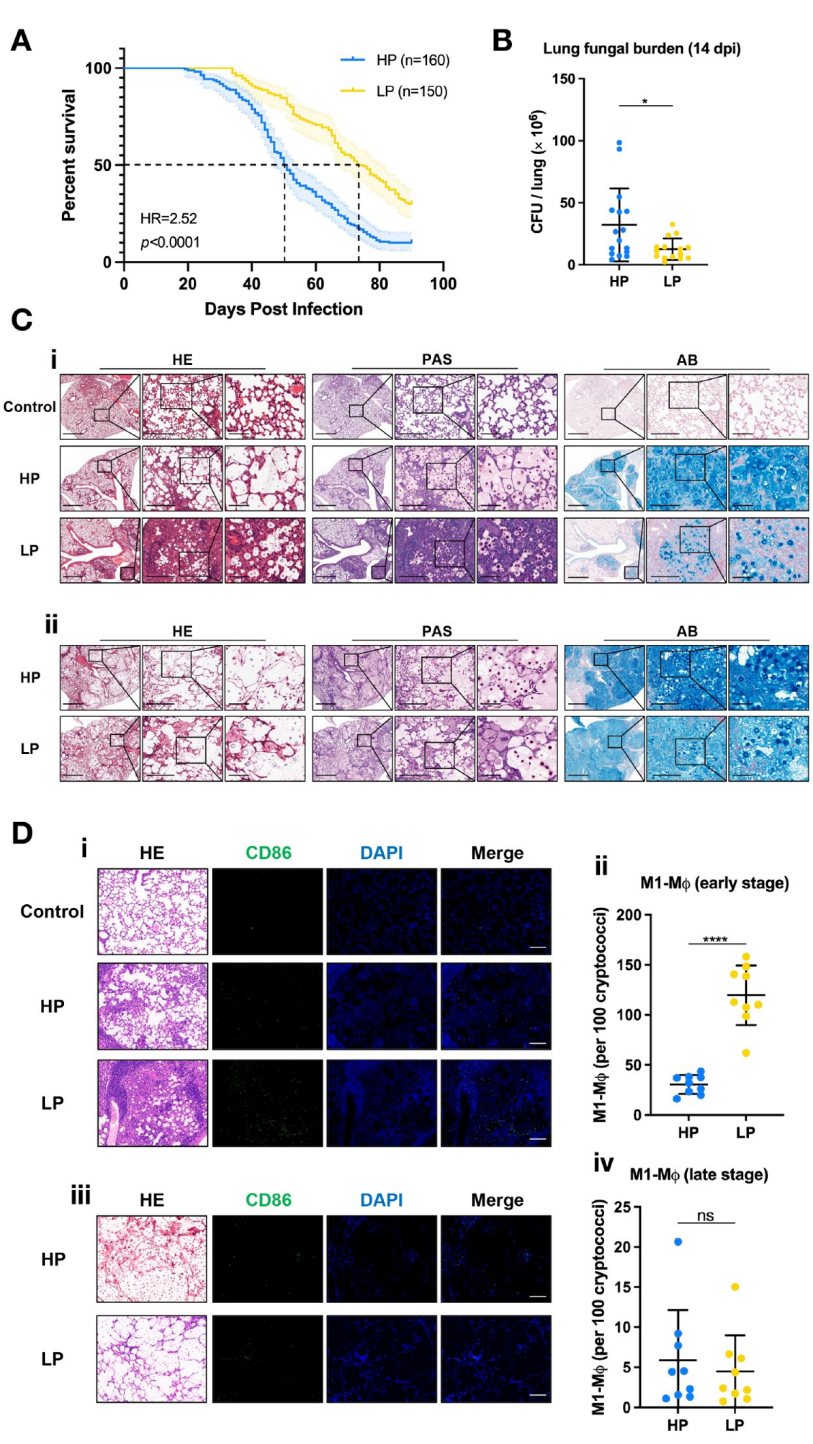
Figure 5 The pulmonary cryptococcal load, pathological structure, number of M1-type macrophages, and virulence differed between the Cg HP and LP strains at the early stage of infection (A) A significant difference was shown in the survival curve of mice infected with the Cg HP and LP strains (P<0.0001). Ten mice per strain group were used in the survival test. The shadow of the survival curves represents the 95% confidence interval. HR, hazard ratio. (B) A significant difference existed in the lung cryptococcal burden of early infection between the HP and LP groups (p=0.0330). Five mice per isolate were tested. The results were from different isolates of each phagocytosis group, and all values are presented as the mean±SD. *P<0.05. (C). Three mice at each stage (14 dpi and death or up to 90 dpi) infected with each isolate from the HP and LP groups were sacrificed and subjected to lung pathological sections. Adjacent sections were stained with HE, PAS, and AB. The images on display were from experiments conducted with similar results. Scale bars, 1000 μm (left panels), 200 μm (middle panels), and 50 μm (right panels). dpi, days postinfection. HE, hematoxylin-eosin. PAS, periodic acid-Schiff. AB, Alcian blue. (D) mIF was utilized to observe the distribution of M1-MØ during cryptococcosis. Granulomas surrounding LP contained more M1-MØ than HP at the early stage of infection (P<0.0001, panels i and ii), and no significant difference was observed at the late stage (P=0.6048, panels iii and iv). The images shown above were from experiments conducted with similar results. All values are presented as the mean±SD. Scale bar: 100 μm. ****P<0.0001. dpi, days postinfection. HE, hematoxylin-eosin. mIF, multiple immunofluorescence. MØ, macrophage. ns, no significant difference.

To assess whether the phagocytosis phenotypes of Cg are correlated with virulence *in vivo*, we used a mouse model of inhalation infection. According to the survival curve, mice infected with the HP strains started dying on day 19, but those in the LP group did not die until day 34 ( Figure 5A). Although both of the ending survival rates of these two groups were low (<50%), mice in the HP group had a lower early survival rate than those in the LP group ( *P<*0.0001), and the median survival times of the two groups were 50 and 74 days, respectively. These findings suggested that although the ultimate virulence of the two groups was strong, the HP strains caused pathogenicity earlier than LP strains within the host. To verify this hypothesis from the histopathological perspective, lung pathological sections at the early stage (day 14) and the late stage of infection (death or up to day 90) were harvested, and the adjacent sections were stained with hematoxylin-eosin (HE), periodic acid-Schiff (PAS), and Alcian blue (AB). As shown in Figure 5C-i, the lung region of LP cryptococcal infection was relatively limited at the early stage, with most of the alveoli being normal and unaffected. Meanwhile, LP cryptococci triggered a robust immune response of the host, spatially confined within restrictive granulomas, which were primarily composed of macrophages. Conversely, the normal structure of alveoli at the site of HP cryptococcal infection disappeared and was filled with a large amount of mucus and acidic polysaccharides. HP cryptococci were distributed widely in the lungs, with permissive granulomas failing to restrict them. Furthermore, the amounts of HP cryptococci in the lungs were larger than those in the LP group according to the lung cryptococcal burden test at the early stage of infection ( *P*=0.0330; Figure 5B). By the end stage of infection, the normal structure of the lungs was destroyed in both the HP and LP groups, cryptococci were released in significant quantities by lysed macrophages, and the dysfunctional pulmonary alveoli were filled with abundant mucus and acidic polysaccharides ( Figure 5C-ii). It could be observed from the gross anatomical lung specimens that at the late stage, the lungs of mice infected with HP and LP strains both showed obvious edema and pale convex structures on the surface ( Supplementary Figure S4, red arrows). These results suggested that the HP and LP strains both underwent gemmation and caused fatal invasion and destruction of the host lungs during the end stage of infection. HP strains proliferate and impair the host’s respiratory system much earlier, thus exerting virulence earlier than LP strains. Detailed data can be found in the raw data sheet in the Supplementary Material.

### Distribution of M1-type macrophages was observed in Cg-infected murine lungs

M1-type macrophages (M1-MØ) are a classical activated state of macrophages, showing a strong phagocytic and killing effect towards pathogens by producing reactive oxygen species (ROS) and reactive nitrogen species (RNS) [30]. To investigate whether HP and LP strains differed in inducing M1-type macrophages (M1-MØ), we performed immunofluorescence labelling of M1-MØ in lung sections of mice towards CD86, a specific surface marker of M1-MØ [28], at the early stage (14 dpi) and late stage (death or up to 90 dpi) of infection and conducted a semiquantitative analysis. According to Figure 5D-i, the granulomas surrounding the LP contained a large amount of M1-MØ at the early stage of infection, whereas less was induced in the HP group, which was also demonstrated by a semiquantitative analysis ( Figure 5D-ii; *P*<0.0001). Moreover, no significant difference was observed in the amount of M1-MØ between the two groups at the late stage, and the cell amounts were both relatively low ( Figure 5D-iii and 5D-iv; *P*=0.6048). We also found that M1-MØ cells were infrequently observed in the control group (uninfected mice). These results indicated that HP induced fewer M1-MØ than LP at the early stage of infection, avoiding killing by host immunity. Furthermore, both groups significantly reduced the formation of M1-MØs, severely disrupting host immunity at the late stage. More detailed data can be found in the raw data sheet in the Supplementary Material.

## Discussion

Phagocytosis of macrophages in the respiratory system, including alveolar macrophages and those infiltrating the lung interstitium, is an essential mechanism by which the host directly kills *Cryptococcus* invading the respiratory tract and is extremely important for limiting the pathogenicity of *Cryptococcus* and controlling the progression of subsequent diseases. However, in this battle, *Cryptococcus* has been proven to survive and proliferate in macrophages [31], spread outside the lungs [32], and even cross the blood‒brain barrier into the central nervous system by the Trojan horse effect via macrophages [12]. Therefore, further study of the pathogen‒host relationship between *Cryptococcus* and macrophages is helpful for understanding the pathogenesis of this deadly pathogen and the mechanism of its immune escape. However, previous relevant studies mainly focused on Cn and paid less attention to Cg, which appeared to have a lower incidence but caused outbreaks and even infected people with normal immunity. In this study, a series of *in vitro* and *in vivo* experiments were conducted to investigate the mutual interaction between macrophages and Cg.

In this study, we demonstrated that (i) Cg displayed diverse phagocytosis phenotypes within macrophages independent of molecular type; (ii) the intracellular proliferation capacity of HP strains was higher than that of LP strains; and (iii) HP strains caused pathogenicity earlier than LP strains *in vivo*.

The phagocytosis assay showed no difference in the phagocytic index between the VGI and VGII groups, indicating that phagocytosis of Cg had no correlation with molecular type. Furthermore, different phagocytic performances of the HP and LP strains were observed by electron microscopy.

Following phagocytosis, cryptococci are exposed to ROS and RNS, as well as antimicrobial peptides that effectively kill and digest most microorganisms within macrophages [30]. The results of intracellular cryptococcal survival and proliferation assays indicated that HP strains of Cg had a more vital proliferation ability than LP strains within macrophages. In addition, the phagocytic index of the Cg strains was positively correlated with IPR ( Supplementary Figure S3), indicating that HP strains may be better adapted to the harsh immune environment within macrophages, thus having a stronger ability to proliferate intracellularly. May Lab reported that hypervirulent strains of Cg were positively correlated with intracellular proliferative capacity within macrophages [ 16, 33]. Our experiments verified a significant correlation between IPR from *in vitro* experiments and mouse survival data (half-life time, ST50) ( Supplementary Figure S5). Compared to animals infected with high IPR strains, mice infected with strains with low IPR values had better survival (18 hpi, *P*<0.0001, R ^2^=0.7670; 24 hpi, *P*<0.0001, R ^2^=0.6694. Linear regression, *n*=31). Enhanced mitochondrial tubularization of Cg contributed to intracellular proliferation within macrophages, which was a possible species-specific virulence mechanism [ 16, 33]. The characteristics of mitochondrial morphology after intracellular growth within macrophages will be the focus of our future studies. It is an interesting phenomenon that highly pathogenic strains of Cg are more likely to be engulfed rather than resisting engulfment by macrophages. Farrer *et al*. [34] also reported that Cg yeast uptake by macrophages was positively correlated with their intracellular proliferative rate ( *P=*0.004, R ^2^=0.3374. Linear regression, *n*=21) [34], which indicated that the phagocytosis phenotype of Cg is associated with virulence, as IPR has been demonstrated to be an effective indicator of Cg virulence, as mentioned above. However, the mechanism has not been fully revealed, and the related research is relatively limited. In further research, we will investigate the mechanism underlying this intriguing phenomenon and determine why highly virulent strains are more likely to be phagocytosed by macrophages.

Macrophages infiltrating the alveoli are the first line of defense against *Cryptococcus* within the host immune system [9]. Sabiiti *et al*. [11] confirmed that Cn with high uptake via macrophages *in vitro* led to a higher cryptococcal load in target organs and poorer survival *in vivo*, which has been confirmed in many studies [ 14, 35]. To test whether the different phagocytosis phenotypes of Cg *in vitro* cause different pathogenicities *in vivo*, we performed inhalation-infected murine experiments in both groups. Survival and pulmonary cryptococcal load tests revealed that HP strains exhibited earlier pathogenicity than LP strains. Intriguingly, the pathological morphology of granulomas induced by infection differed between the phagocytosis groups. Restrictive granulomas effectively restricted LP strains from intrapulmonary spreading at the early stage, while permissive granulomas failed to limit HP strains. Similar to *Mycobacterium tuberculosis* infection, granuloma is a pathological sign of cryptococcal infection [36]. When pathogens (perhaps in alveolar macrophages or other phagocytes) are transferred into the lung parenchyma, the host initiates an immune response in which monocytes, macrophages, neutrophils, and dendritic cells are attracted to the site of infection, possibly due to chemokine and cytokine signaling from infected cells, triggering the formation of granulomas [ 36, 37]. Inspired by the work of Flynn *et al*. [37], we suggested that HP and LP strains may induce permissive and restrictive granulomas within the host at the early stage, respectively.

M1-type macrophages (M1-MØ) are classically activated by IFN-γ produced by type 1 immune responses via signal transduction and activator of transcription-1 signaling pathway (STAT1), which produce ROS and RNS and show a strong phagocytic effect on pathogens [30]. However, *Cryptococcus* has been proven to inhibit the formation of M1-MØs by inducing the polarization of macrophages towards the nonclassical activation type via the Hsp 70 homolog Ssa1, as well as impairing the killing of macrophages [ 38, 39]. We found that HP strains induced significantly fewer M1-MØ than LP strains at the early stage of infection, and large amounts of cryptococci occupied pulmonary alveoli with abundant mucus and acidic polysaccharides. Thus, we suspected that HP strains may have a stronger ability to inhibit the formation of M1-MØs, achieving intracellular proliferation and extracellular dissemination at the early stage. This might explain the pathogenic difference at the early stage between the Cg isolates with different phagocytosis phenotypes.

**Figure 6:**
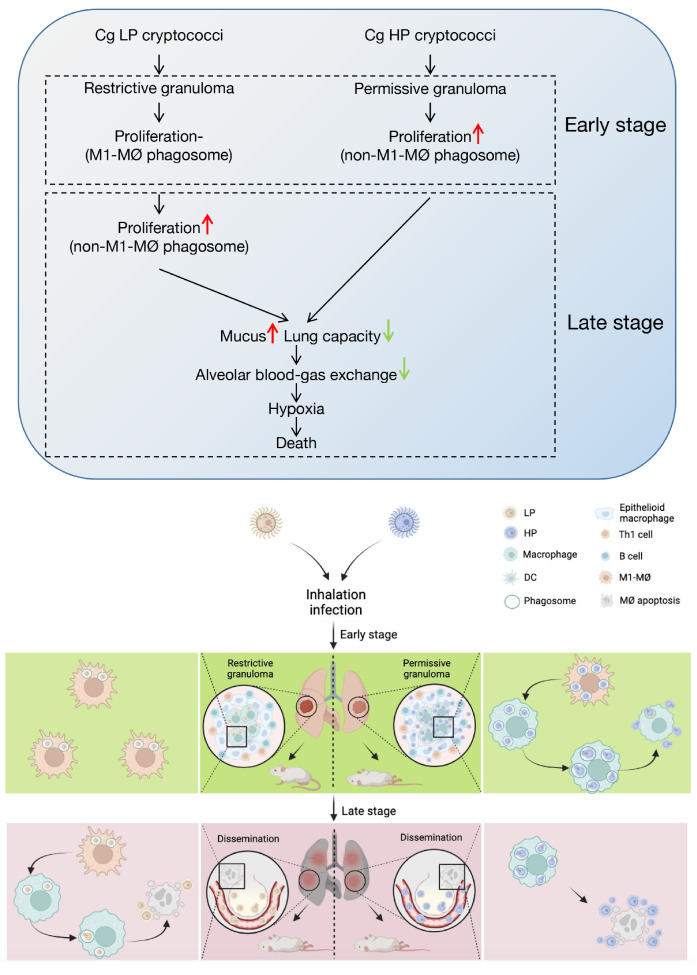
Figure 6 Proposed model depicting Cg HP and LP strains’ different pathogenicity *in vivo* Mice were inhalation-infected with Cg cryptococci. At the early stage of infection, restrictive granulomas formed in the LP group lungs, and cryptococcal proliferation was limited within M1-type macrophages, while HP-inducing permissive granulomas seldom limited the proliferation of pathogens. At the cellular level, HP induced MØ from the M1-type to the nonclassical activation type and proliferated within phagosomes. After that, HP strains escaped from macrophages and spread rapidly in the pulmonary interstitium, generating abundant mucus and acidic polysaccharide substances and causing death at the early stage. At the late stage, the HP and LP strains were both able to disseminate extrapulmonary via vascular transportation. LP strains also completed MØ induction and proliferated quickly, causing macrophage apoptosis. Hypoxia caused by poor alveolar blood-gas exchange with mucus accumulation and diminished lung capacity may be the leading cause of death in the HP and LP groups at the late stage. LP, low phagocytosis phenotype. HP, high phagocytosis phenotype. “‒”, normal level. MØ, macrophage. DC, dendritic cells. Created in BioRender.com.

When infected for a sufficiently long time, the LP strains could also induce the transformation of macrophages from M1-type to non-M1-type and proliferate within phagosomes. Furthermore, both HP and LP cryptococci escape from lysed macrophages in large quantities and occupy pulmonary alveoli. The typical alveolar structure is destroyed, with abundant mucus and acidic polysaccharide substances accumulating. Liu *et al*. [40] demonstrated that mucins accumulate in the bronchoalveolar lavage fluid (BALF) of patients with coronavirus disease 2019 (COVID-19), and consequently, alveolar mucus affects the blood-gas barrier, thus inducing hypoxia and diminishing lung capacity. A clinic-based study found that mucus and emphysema have a direct correlation with airflow limitation and hypoxemia [41]. Inspired by the discovery above and several clinical cases of pneumonia caused by other pathogens [ 42, 43], we believe that at the late stage of cryptococcal infection, in addition to the traditional virulence factors of *Cryptococcus* itself (capsule, melanin, 37°C growth ability, etc.), hypoxia caused by poor alveolar blood-gas exchange due to mucus and the loss of effective lung respiratory units may be the main cause of death [ 40‒ 43], which might also shed light on the early pathogenicity *in vivo* caused by Cg HP strains. Survival analysis also confirmed that although the HP and LP strains both induced poor prognoses at the late stage, the HP strains showed pathogenicity earlier *in vivo*. However, more extensive studies, including strains from other geographical regions with different types of infection models, should be carried out to support our findings. We tried to explain the different pathogenicities and possible mechanisms of HP and LP strains in Figure 6.

In summary, through *in vitro* and *in vivo* studies, we found that the phagocytosis phenotypes of Cg were molecular-type-independent, and HP cryptococci obtained a stronger intracellular proliferation capacity within macrophages. Both the HP and LP strains showed high virulence, while the HP strains were more virulent at the early stage. This may lead us to pay additional attention to Cg pathogenicity in early infection and early clinical treatments. Nevertheless,, there were still some limitations in our study, such as the lack of verification of our findings with more macrophage lines and the lack of further exploration of whether a certain class of regulatory genes is in charge of the differential phagocytosis phenotypes of HP and LP strains. Our current results and experiment-based hypotheses nevertheless shed light on the field of Cg-macrophage interactions. Recently, Denham *et al*. [32] found that smaller forms of Cn are more susceptible to phagocytosis by macrophages and extrapulmonary dissemination. Whether our Cg HP strains obtain a smaller size and therefore have stronger phagocytosis within macrophages and pathogenicity within the host needs to be further investigated in future studies.

## Supporting information

Supplementary

Supplementary

## Supplementary Data

Supplementary data is available at *Acta Biochimica et Biophysica Sinica*.
