# Supplementary material for: Cryptococcus gattii strains with a high phagocytosis phenotype by macrophages display high pathogenicity at the early stage of infection in vivo : High phagocytosis Cg displays early pathogenicity
Source: Acta Biochim Biophys Sin (Shanghai). 2023 Oct 26;56(2):291–303. doi: 10.3724/abbs.2023250 (PMC10984874; doi:10.3724/abbs.2023250)
Supplement: Supplementary [file Supplementary.pdf]

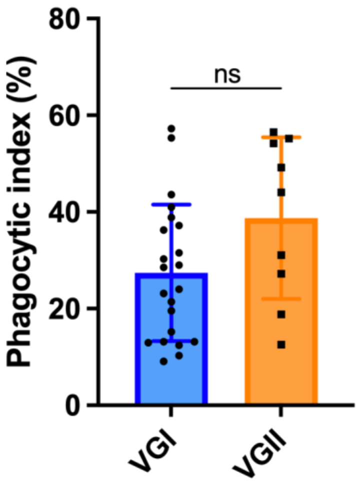

Supplementary Figure S1. Comparation of the phagocytic index (PI) between *Cryptococcus gattii* (Cg) VGI and VGII strains We compared PI according to the molecular types of Cg, and no significant difference was observed between VGI and VGII (*P*=0.1113). The results are representative of three independent replicates.

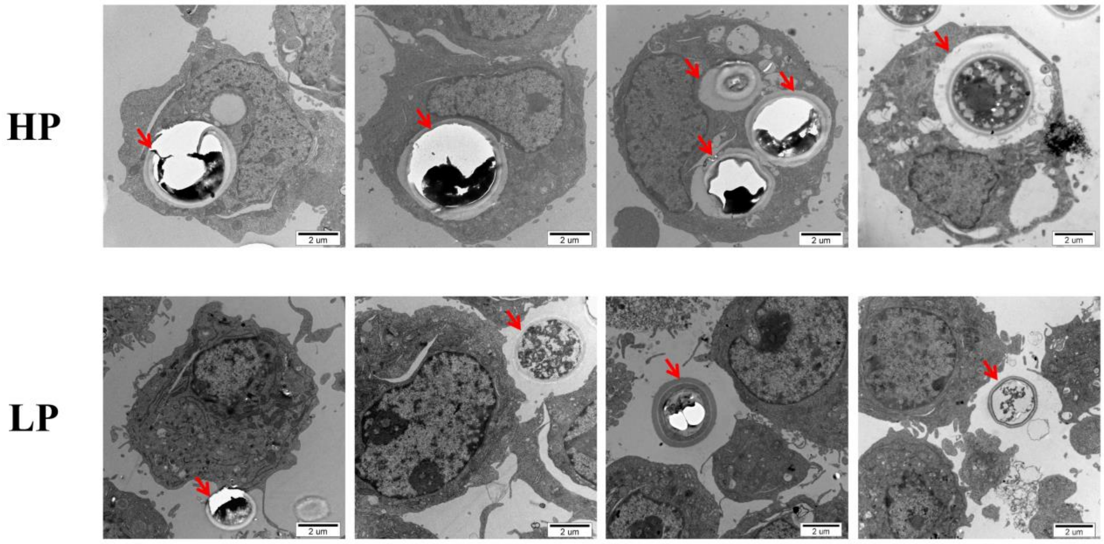

Supplementary Figure S2. TEM images of Cg strains with diverse phagocytosis phenotypes and macrophages at 2-h co-incubation Additional TEM images of 2-h interactions between the HP and LP groups are illustrated. The HP and LP strains displayed high and low phagocytosis phenotypes within macrophages, respectively. Cryptococci were indicated by red arrows. Scale bar: 2 µm. Transmission electron microscopy (TEM).

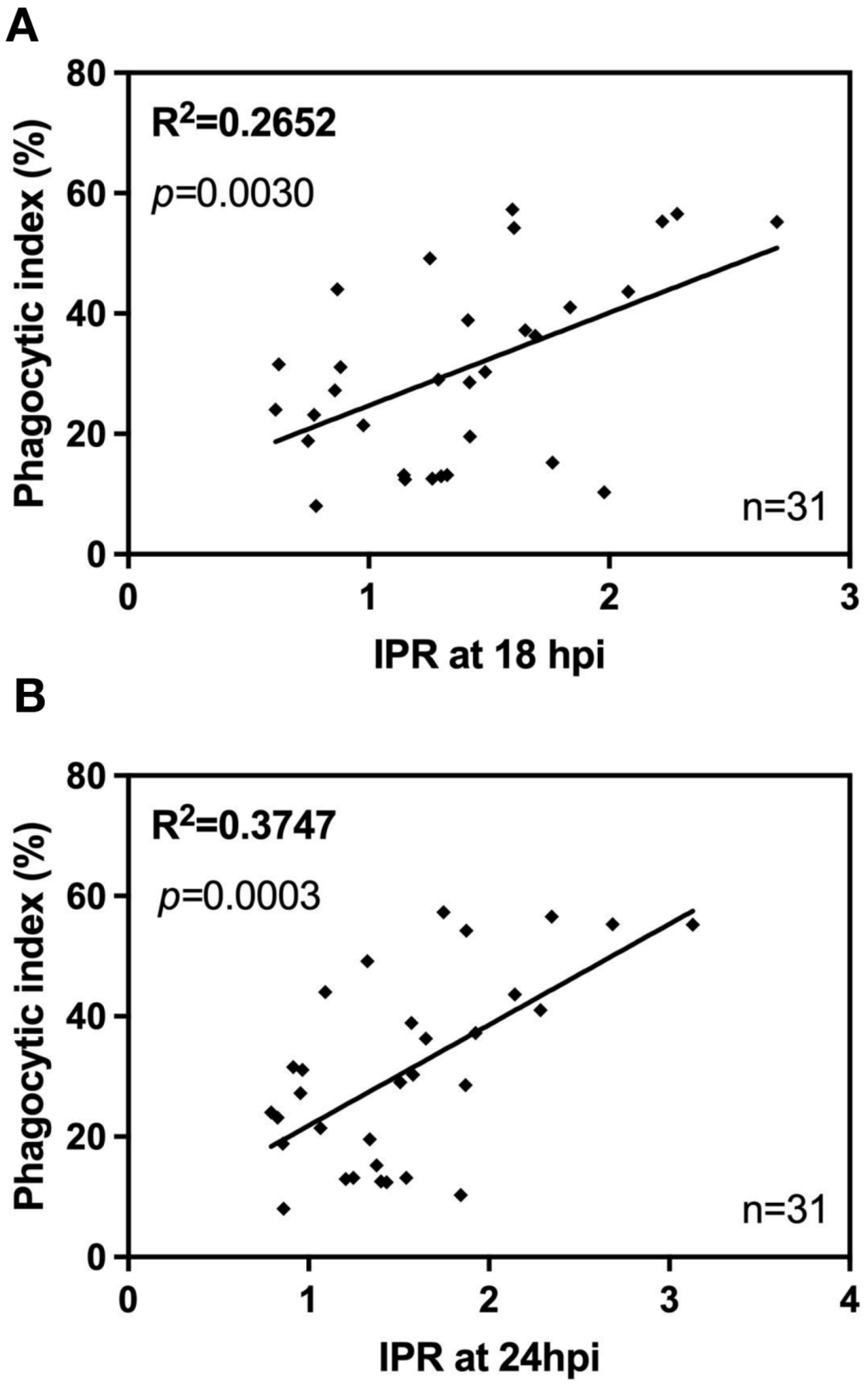

Supplementary Figure S3. A significant correlation between intracellular proliferation rate (IPR) and phagocytic index (PI) Cg strains with higher PI had a stronger capacity of proliferation within macrophages than lower ones. (**A**, 18 hpi, R^2=0.2652, *P*=0.0030; **B**, 24 hpi, R^2=0.3747,

P=0.0003. Linear regression, *n*=31). Refer to raw data sheet in supplemental material for detailed data.

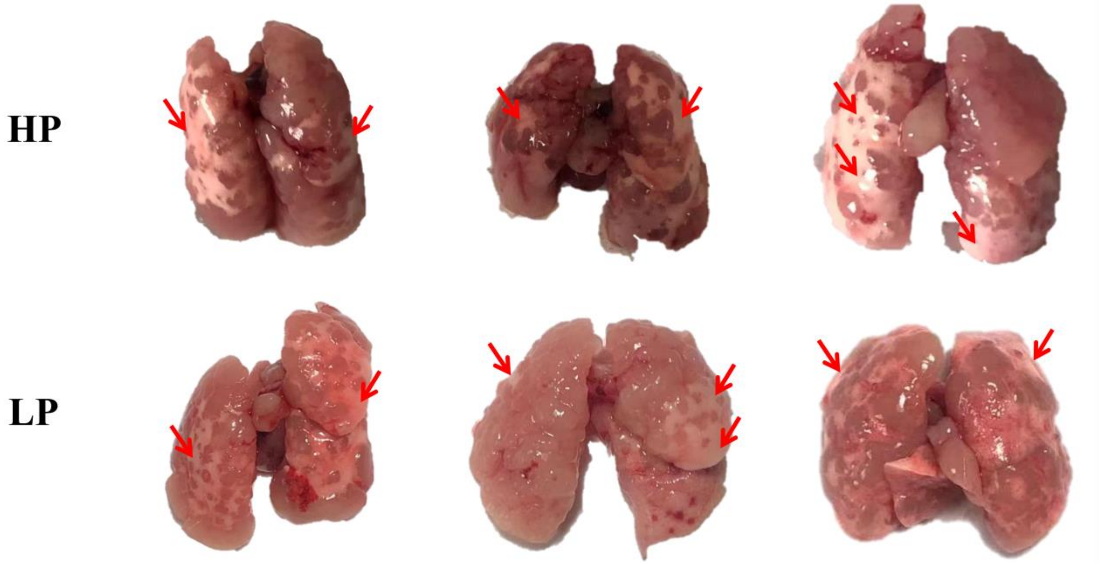

Supplementary Figure S4. Gross specimens of lungs at the late stage of infection dissected from HP and LP strains-infected mice Both the HP and LP groups showed obvious edema of the lungs and pale convex structures formed on the lung surface (indicated by red arrows).

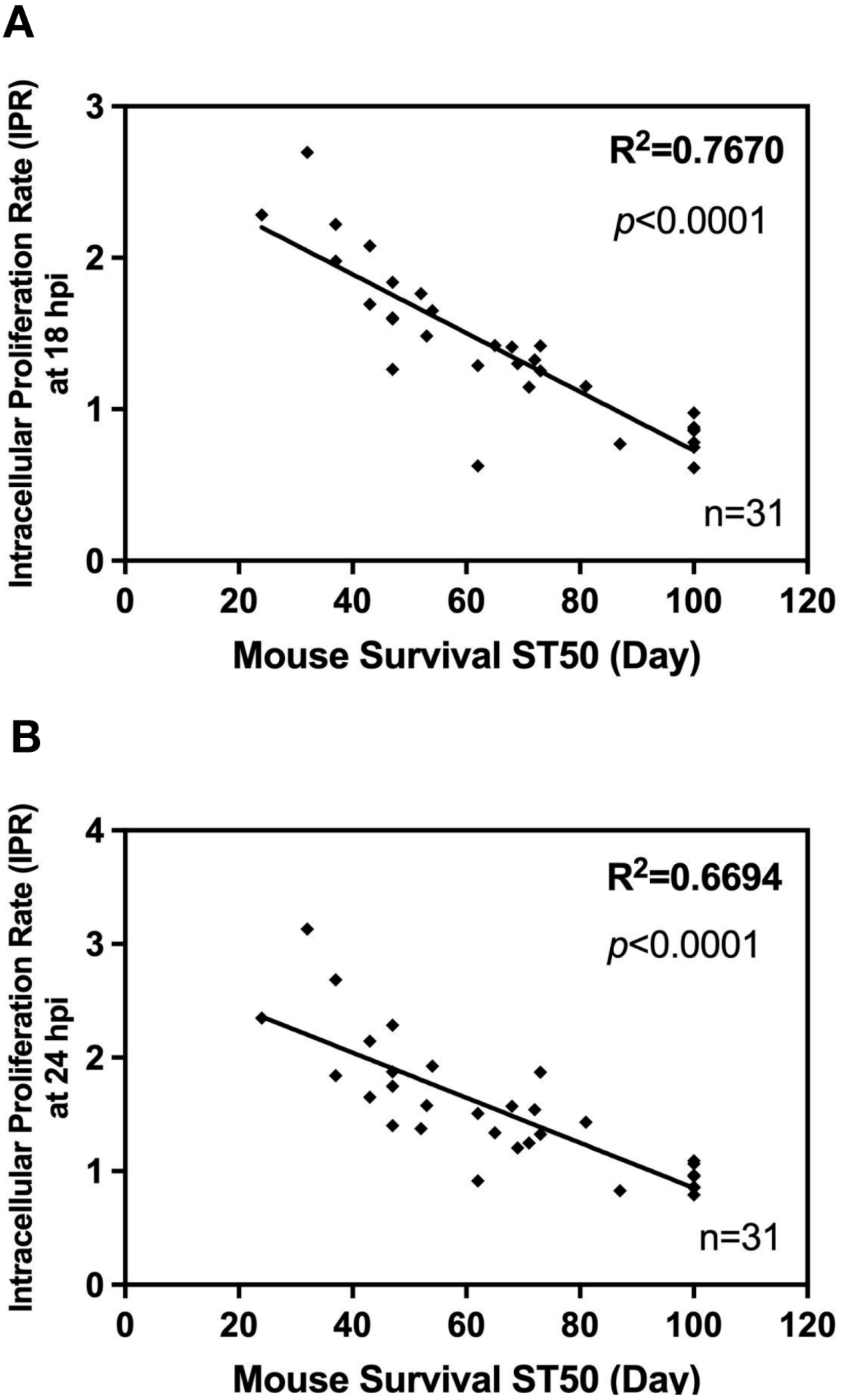

Supplementary Figure S5. A significant correlation between mouse survival data and intracellular proliferation rate (IPR) Mice survived longer when infected with strains with low

IPR values than when infected with high IPR strains (**A**, 18 hpi, R^2=0.7670, *P*<0.0001; **B**, 24 hpi, R^2=0.6694, *P*<0.0001. Linear regression, *n*=31). We arbitrarily assigned an ST50 of 100 days for strains that did not result in a mortality of 50% or higher within the 90-day time frame of the experiment. Refer to the raw data sheet in the supplemental material for the detailed data.

Supplementary Table S1. Phagocytic index of 31 *Cryptococcus gattii* strains by flow cytometry

Strain ID	Genotype	Phagocytic index (%) (mean ± SD)
G2	VGI	57.27 ± 0.57
R265	VGII	56.53 ± 0.49
WM276	VGI	55.30 ± 0.50
G14	VGII	55.07 ± 0.65
G10	VGII	54.20 ± 0.95
G6	VGII	49.16 ± 0.95
R272	VGII	44.03 ± 0.91
G9	VGI	43.60 ± 0.95
G15	VGI	41.00 ± 1.81
G24	VGI	38.90 ± 1.83
G18	VGI	37.20 ± 0.89
G20	VGI	36.30 ± 0.46
G8	VGI	31.53 ± 1.32
G13	VGII	31.10 ± 1.73
G4	VGI	30.30 ± 0.95
G16	VGI	29.00 ± 1.45
G19	VGI	28.53 ± 1.10
G26	VGII	27.20 ± 0.75
G7	VGI	24.00 ± 1.11
G5	VGI	23.13 ± 1.79
G21	VGI	21.43 ± 1.04
G3	VGI	19.53 ± 0.29
G17	VGII	18.80 ± 0.62
G28	VGI	15.23 ± 0.30
G12	VGI	13.13 ± 0.47
G29	VGI	13.13 ± 0.30
G23	VGI	12.93 ± 0.51
G1	VGII	12.53 ± 0.30
G30	VGI	12.40 ± 0.43
G25	VGI	10.27 ± 0.58
G27	VGI	9.07 ± 1.00

The phagocytic index results of the 31 Cg strains by flow cytometry were listed. Strains with a

phagocytic index greater than the median value (29%) were defined as the high phagocytic index group (HP), and the rest strains were defined as the low phagocytic index group (LP). All data are representative of three independent replicate tests.
